# Bis(3,5-dinitro­benzoato-κ*O* ^1^)tetra­methano­lcobalt(II)

**DOI:** 10.1107/S1600536810007580

**Published:** 2010-03-06

**Authors:** Bipasa Sarkar, Abhijit Roy, Seik Weng Ng, Edward R. T. Tiekink

**Affiliations:** a Department of Chemistry, North Bengal University, Darjeeling, West Bengal 734 430, India; b Department of Chemistry, University of Malaya, 50603 Kuala Lumpur, Malaysia

## Abstract

The Co^II^ atom (site symmetry 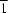) in the title complex, [Co(C_7_H_3_N_2_O_6_)_2_(CH_3_OH)_4_], exists within an octa­hedral O_6_ donor set defined by two *O*-monodentate 3,5-dinitro­benzoate anions and four methanol O atoms. An intra­molecular O_m_—H⋯O_c_ (m = methanol and c = carbon­yl) hydrogen bond leads to the formation of an *S*(6) ring. In the crystal, centrosymmetrically related mol­ecules associate *via* further O_m_—H⋯O_c_ hydrogen bonds, leading to linear supra­molecular chains propagating along the *a*-axis direction.

## Related literature

For the structures of related complexes, see: Tahir *et al.* (1996 ▶); Yang *et al.* (2000 ▶); Jin *et al.* (2008 ▶). For a description of the Cambridge Structural Database, see: Allen (2002 ▶).
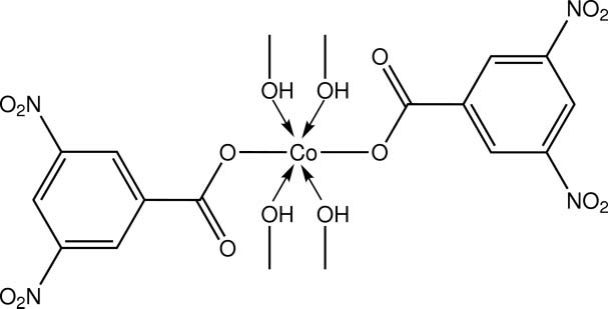


## Experimental

### 

#### Crystal data

[Co(C_7_H_3_N_2_O_6_)_2_(CH_4_O)_4_]
*M* *_r_* = 609.33
Triclinic, 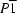

*a* = 6.4068 (8) Å
*b* = 8.7660 (11) Å
*c* = 12.1603 (16) Å
α = 90.411 (2)°
β = 100.407 (2)°
γ = 102.214 (2)°
*V* = 655.77 (14) Å^3^
*Z* = 1
Mo *K*α radiation
μ = 0.74 mm^−1^
*T* = 293 K
0.35 × 0.30 × 0.05 mm

#### Data collection

Bruker SMART APEX diffractometer
Absorption correction: multi-scan (*SADABS*; Sheldrick, 1996 ▶) *T*_min_ = 0.669, *T*_max_ = 0.746
6372 measured reflections
2999 independent reflections
2388 reflections with *I* > 2σ(*I*)
*R*_int_ = 0.028

#### Refinement

*R*[*F* ^2^ > 2σ(*F* ^2^)] = 0.043
*wR*(*F* ^2^) = 0.110
*S* = 1.03
2999 reflections
188 parameters
2 restraints
H atoms treated by a mixture of independent and constrained refinement
Δρ_max_ = 0.38 e Å^−3^
Δρ_min_ = −0.30 e Å^−3^

### 

Data collection: *APEX2* (Bruker, 2008 ▶); cell refinement: *SAINT* (Bruker, 2008 ▶); data reduction: *SAINT*; program(s) used to solve structure: *SHELXS97* (Sheldrick, 2008 ▶); program(s) used to refine structure: *SHELXL97* (Sheldrick, 2008 ▶); molecular graphics: *ORTEP-3* (Farrugia, 1997 ▶) and *DIAMOND* (Brandenburg, 2006 ▶); software used to prepare material for publication: *publCIF* (Westrip, 2010 ▶).

## Supplementary Material

Crystal structure: contains datablocks global, I. DOI: 10.1107/S1600536810007580/hb5347sup1.cif

Structure factors: contains datablocks I. DOI: 10.1107/S1600536810007580/hb5347Isup2.hkl

Additional supplementary materials:  crystallographic information; 3D view; checkCIF report

## Figures and Tables

**Table 1:** Selected bond lengths (Å)

Co—O8	2.0645 (18)
Co—O1	2.0666 (17)
Co—O7	2.1094 (16)

**Table 2:** Hydrogen-bond geometry (Å, °)

*D*—H⋯*A*	*D*—H	H⋯*A*	*D*⋯*A*	*D*—H⋯*A*
O7—H7o⋯O2^i^	0.85 (3)	1.84 (2)	2.645 (2)	158 (3)
O8—H8o⋯O2^ii^	0.85 (3)	1.82 (1)	2.662 (2)	179 (3)

Symmetry codes: (i) 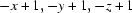; (ii) 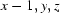.

**Fig. 1.**
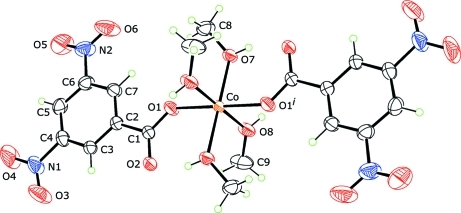
The molecular structure of (I) extended to show the coordination geometry for the Co(II) atom, showing displacement ellipsoids at the 50% probability level. Symmetry operation i: 1-x, 1-y, 1-z.

**Fig. 2.**
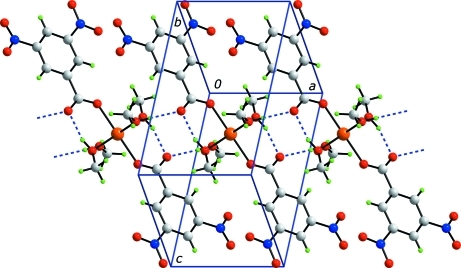
Linear supramolecular chain along the a axis in (I) mediated by O—H···O hydrogen bonding. These and the intramolecular O–H···O hydrogen bonds are shown as blue dashed lines.

## supplementary crystallographic information

### Comment

The Co(II) atom in (I), Fig. 1, is located on a crystallographic centre of inversion and exists within an octahedral O_6_ donor set defined by two carboxylate-O1 atoms and four methanol-O atoms. The Co–O1 bond distance [Co–O1 = 2.0666 (17) Å] is comparable to those, *i*.*e*. 2.0525 (20) and 2.0587 (19) Å, found in the related tetra-aqua-bis(3,5-dinitrobenzoato-*O*)cobalt(II) tetrahydrate structure (Tahir *et al.*, 1996; Yang *et al.*, 2000; Jin *et al.*, 2008). A small disparity in the Co—O_methanol_ bond distances in (I) [Co–O7 = 2.1094 (16) and Co–O8 = 2.0645 (18) Å] is noted. The methanol-O7–H hydrogen forms an intramolecular O–H···O hydrogen bond with the carbonyl-O2 atom to close an almost planar {Co–O–C–O···H–O} S(6) ring, Table 1. The methanol-O8–H also forms a hydrogen bond to the carbonyl-O2 atom on a centrosymmetrically related complex, Table 1. This results in the formation of 12-membered {Co–O–H···O–C–O}_2_ synthons and linear supramolecular chains along the *a* axis, Fig. 2. It is noted that the packing of molecules brings into close proximity two nitro-O atoms, i.e. O4···O4^ii^ = 2.756 (3) Å for 3-*x*, -*y*, 2-*z*. While the nature of this interaction is not obvious, there are approximately 50 precedents for such O_nitro_···O_nitro_ contacts < 2.70 Å in the crystallographic literature (Allen, 2002).

### Experimental

All chemicals purchased from commercial sources (AR/GR grade) were used without further purification. The crystalline sodium salt of 3,5-dinitrobenzoic acid was prepared by neutralising 3,5-dinitrobenzoic acid (1.00 g, 4.70 mmol) by NaOH (1.88 g, 4.70 mmol) in water. On concentration the solution yielded the sodium salt (1). To a purple solution of CoCl_2_^.^6H_2_O (0.50 g, 2.10 mmol) in water (15 ml) was added, with stirring, a solution of (1) (0.98 g, 4.20 mmol) in a mixture of water (3 ml) and methanol (140 ml) when the colour of the mixture turned pink. The solution was then heated under reflux for 6 h. Pink prisms of (I) were obtained from the filtrate after 25 days on slow evaporation.

### Refinement

Carbon-bound H-atoms were placed in calculated positions (C—H 0.93 to 0.96 Å) and were included in the refinement in the riding model approximation, with *U*_iso_(H) set to 1.2 to 1.5*U*_equiv_(C). The methanol H-atoms were located in a difference Fourier map, and were refined with a distance restraint of O–H 0.85±0.01 Å; their *U_iso_* values were freely refined

### Crystal data

**Table:**

[Co(C_7_H_3_N_2_O_6_)_2_(CH_4_O)_4_]	*Z* = 1
*M**_r_* = 609.33	*F*(000) = 313
Triclinic, *P*1	*D*_x_ = 1.543 Mg m^−^^3^
Hall symbol: -P 1	Mo *K*α radiation, λ = 0.71073 Å
*a* = 6.4068 (8) Å	Cell parameters from 1592 reflections
*b* = 8.7660 (11) Å	θ = 2.4–22.8°
*c* = 12.1603 (16) Å	µ = 0.74 mm^−^^1^
α = 90.411 (2)°	*T* = 293 K
β = 100.407 (2)°	Prism, pink
γ = 102.214 (2)°	0.35 × 0.30 × 0.05 mm
*V* = 655.77 (14) Å^3^	

### Data collection

**Table:**

Bruker SMART APEX diffractometer	2999 independent reflections
Radiation source: fine-focus sealed tube	2388 reflections with *I* > 2σ(*I*)
graphite	*R*_int_ = 0.028
ω scans	θ_max_ = 27.5°, θ_min_ = 1.7°
Absorption correction: multi-scan (*SADABS*; Sheldrick, 1996)	*h* = −8→8
*T*_min_ = 0.669, *T*_max_ = 0.746	*k* = −11→11
6372 measured reflections	*l* = −15→15

### Refinement

**Table:**

Refinement on *F*^2^	Primary atom site location: structure-invariant direct methods
Least-squares matrix: full	Secondary atom site location: difference Fourier map
*R*[*F*^2^ > 2σ(*F*^2^)] = 0.043	Hydrogen site location: inferred from neighbouring sites
*wR*(*F*^2^) = 0.110	H atoms treated by a mixture of independent and constrained refinement
*S* = 1.03	*w* = 1/[σ^2^(*F*_o_^2^) + (0.0522*P*)^2^ + 0.1427*P*] where *P* = (*F*_o_^2^ + 2*F*_c_^2^)/3
2999 reflections	(Δ/σ)_max_ = 0.001
188 parameters	Δρ_max_ = 0.38 e Å^−^^3^
2 restraints	Δρ_min_ = −0.30 e Å^−^^3^

### Special details

**Table:**

Geometry. All s.u.'s (except the s.u. in the dihedral angle between two l.s. planes) are estimated using the full covariance matrix. The cell s.u.'s are taken into account individually in the estimation of s.u.'s in distances, angles and torsion angles; correlations between s.u.'s in cell parameters are only used when they are defined by crystal symmetry. An approximate (isotropic) treatment of cell s.u.'s is used for estimating s.u.'s involving l.s. planes.
Refinement. Refinement of *F*^2^ against ALL reflections. The weighted *R*-factor wR and goodness of fit *S* are based on *F*^2^, conventional *R*-factors *R* are based on *F*, with *F* set to zero for negative *F*^2^. The threshold expression of *F*^2^ > 2σ(*F*^2^) is used only for calculating *R*-factors(gt) etc. and is not relevant to the choice of reflections for refinement. *R*-factors based on *F*^2^ are statistically about twice as large as those based on *F*, and *R*- factors based on ALL data will be even larger.

### Fractional atomic coordinates and isotropic or equivalent isotropic displacement parameters (Å^2^)

**Table:**

	*x*	*y*	*z*	*U*_iso_*/*U*_eq_	
Co	0.5000	0.5000	0.5000	0.03530 (16)	
O1	0.6891 (3)	0.3924 (2)	0.61732 (14)	0.0514 (5)	
O2	0.8795 (3)	0.2811 (2)	0.51696 (14)	0.0471 (4)	
O3	1.4087 (5)	0.0083 (4)	0.7142 (2)	0.1213 (12)	
O4	1.4957 (5)	0.0210 (4)	0.8875 (2)	0.1234 (12)	
O5	1.0327 (5)	0.2304 (4)	1.1038 (2)	0.1159 (11)	
O6	0.8093 (6)	0.3608 (4)	1.0193 (2)	0.1090 (10)	
O7	0.3342 (3)	0.5533 (2)	0.62522 (14)	0.0458 (4)	
H7O	0.255 (4)	0.614 (3)	0.595 (2)	0.075 (11)*	
O8	0.2673 (3)	0.2953 (2)	0.46261 (18)	0.0550 (5)	
H8O	0.145 (3)	0.291 (4)	0.481 (3)	0.075 (10)*	
N1	1.3845 (5)	0.0471 (3)	0.8033 (2)	0.0710 (8)	
N2	0.9450 (5)	0.2836 (4)	1.0197 (2)	0.0775 (8)	
C1	0.8312 (4)	0.3181 (3)	0.60687 (19)	0.0379 (5)	
C2	0.9496 (4)	0.2659 (3)	0.7142 (2)	0.0383 (5)	
C3	1.1103 (4)	0.1830 (3)	0.7114 (2)	0.0420 (6)	
H3	1.1468	0.1592	0.6436	0.050*	
C4	1.2149 (4)	0.1366 (3)	0.8100 (2)	0.0498 (6)	
C5	1.1687 (5)	0.1679 (3)	0.9125 (2)	0.0547 (7)	
H5	1.2420	0.1356	0.9783	0.066*	
C6	1.0075 (5)	0.2500 (3)	0.9125 (2)	0.0525 (7)	
C7	0.8985 (4)	0.2998 (3)	0.8158 (2)	0.0468 (6)	
H7	0.7914	0.3558	0.8191	0.056*	
C8	0.4162 (5)	0.5914 (4)	0.7407 (2)	0.0604 (8)	
H8A	0.3034	0.6156	0.7755	0.091*	
H8B	0.5356	0.6803	0.7491	0.091*	
H8C	0.4652	0.5041	0.7755	0.091*	
C9	0.2797 (6)	0.1460 (3)	0.4244 (3)	0.0787 (11)	
H9A	0.2018	0.1255	0.3487	0.118*	
H9B	0.2166	0.0686	0.4712	0.118*	
H9C	0.4292	0.1422	0.4271	0.118*	

### Atomic displacement parameters (Å^2^)

**Table:**

	*U*^11^	*U*^22^	*U*^33^	*U*^12^	*U*^13^	*U*^23^
Co	0.0357 (3)	0.0419 (3)	0.0339 (3)	0.01829 (19)	0.00943 (18)	0.00124 (18)
O1	0.0558 (11)	0.0699 (12)	0.0409 (10)	0.0385 (10)	0.0118 (8)	0.0069 (8)
O2	0.0428 (9)	0.0662 (12)	0.0387 (9)	0.0257 (8)	0.0080 (7)	0.0001 (8)
O3	0.151 (3)	0.177 (3)	0.083 (2)	0.133 (3)	0.0311 (18)	0.0207 (19)
O4	0.125 (2)	0.193 (3)	0.086 (2)	0.117 (2)	0.0082 (17)	0.044 (2)
O5	0.157 (3)	0.168 (3)	0.0389 (14)	0.074 (2)	0.0149 (15)	0.0175 (16)
O6	0.167 (3)	0.132 (2)	0.0629 (16)	0.085 (2)	0.0510 (17)	0.0109 (15)
O7	0.0501 (11)	0.0574 (11)	0.0379 (9)	0.0259 (9)	0.0125 (8)	0.0023 (8)
O8	0.0428 (11)	0.0497 (11)	0.0767 (13)	0.0086 (9)	0.0242 (10)	−0.0125 (9)
N1	0.0722 (17)	0.088 (2)	0.0672 (18)	0.0498 (16)	0.0113 (14)	0.0207 (15)
N2	0.109 (2)	0.086 (2)	0.0466 (16)	0.0382 (18)	0.0211 (15)	0.0030 (14)
C1	0.0346 (12)	0.0411 (13)	0.0400 (13)	0.0133 (10)	0.0064 (10)	0.0019 (10)
C2	0.0375 (12)	0.0384 (12)	0.0398 (13)	0.0105 (10)	0.0064 (10)	0.0032 (10)
C3	0.0429 (13)	0.0482 (14)	0.0377 (13)	0.0173 (11)	0.0060 (10)	0.0027 (10)
C4	0.0463 (14)	0.0495 (15)	0.0560 (16)	0.0200 (12)	0.0046 (12)	0.0081 (12)
C5	0.0603 (17)	0.0605 (17)	0.0422 (15)	0.0184 (14)	−0.0003 (12)	0.0095 (12)
C6	0.0645 (17)	0.0566 (16)	0.0380 (14)	0.0176 (14)	0.0080 (12)	0.0011 (12)
C7	0.0509 (15)	0.0488 (14)	0.0458 (14)	0.0205 (12)	0.0107 (11)	0.0031 (11)
C8	0.078 (2)	0.0658 (19)	0.0409 (15)	0.0215 (16)	0.0146 (14)	−0.0014 (13)
C9	0.084 (2)	0.0532 (18)	0.104 (3)	0.0050 (17)	0.044 (2)	−0.0217 (18)

### Geometric parameters (Å, °)

**Table:**

Co—O8	2.0645 (18)	N2—C6	1.476 (4)
Co—O8^i^	2.0645 (18)	C1—C2	1.511 (3)
Co—O1	2.0666 (17)	C2—C7	1.380 (3)
Co—O1^i^	2.0666 (16)	C2—C3	1.385 (3)
Co—O7	2.1094 (16)	C3—C4	1.373 (3)
Co—O7^i^	2.1094 (16)	C3—H3	0.9300
O1—C1	1.250 (3)	C4—C5	1.371 (4)
O2—C1	1.247 (3)	C5—C6	1.378 (4)
O3—N1	1.179 (4)	C5—H5	0.9300
O4—N1	1.190 (3)	C6—C7	1.379 (4)
O5—N2	1.220 (4)	C7—H7	0.9300
O6—N2	1.209 (4)	C8—H8A	0.9600
O7—C8	1.418 (3)	C8—H8B	0.9600
O7—H7O	0.85 (3)	C8—H8C	0.9600
O8—C9	1.408 (3)	C9—H9A	0.9600
O8—H8O	0.85 (3)	C9—H9B	0.9600
N1—C4	1.482 (3)	C9—H9C	0.9600
			
O8—Co—O1	91.36 (8)	C7—C2—C3	119.3 (2)
O8^i^—Co—O1	88.64 (8)	C7—C2—C1	120.5 (2)
O8—Co—O1^i^	88.64 (8)	C3—C2—C1	120.2 (2)
O8^i^—Co—O1^i^	91.36 (8)	C4—C3—C2	119.1 (2)
O8—Co—O7	88.14 (7)	C4—C3—H3	120.5
O8^i^—Co—O7	91.86 (7)	C2—C3—H3	120.5
O1—Co—O7	89.22 (7)	C5—C4—C3	123.3 (2)
O1^i^—Co—O7	90.78 (7)	C5—C4—N1	119.3 (2)
O8—Co—O7^i^	91.86 (7)	C3—C4—N1	117.4 (2)
O8^i^—Co—O7^i^	88.14 (7)	C4—C5—C6	116.3 (2)
O1—Co—O7^i^	90.78 (7)	C4—C5—H5	121.9
O1^i^—Co—O7^i^	89.22 (7)	C6—C5—H5	121.9
O1—Co—O1^i^	180.0	C5—C6—C7	122.6 (2)
O7—Co—O7^i^	180.0	C5—C6—N2	119.1 (3)
O8—Co—O8^i^	180.0	C7—C6—N2	118.3 (3)
C1—O1—Co	130.70 (16)	C6—C7—C2	119.4 (2)
C8—O7—Co	128.82 (16)	C6—C7—H7	120.3
C8—O7—H7O	113 (2)	C2—C7—H7	120.3
Co—O7—H7O	105 (2)	O7—C8—H8A	109.5
C9—O8—Co	131.57 (18)	O7—C8—H8B	109.5
C9—O8—H8O	110 (2)	H8A—C8—H8B	109.5
Co—O8—H8O	118 (2)	O7—C8—H8C	109.5
O3—N1—O4	122.3 (3)	H8A—C8—H8C	109.5
O3—N1—C4	118.6 (3)	H8B—C8—H8C	109.5
O4—N1—C4	119.1 (3)	O8—C9—H9A	109.5
O6—N2—O5	123.7 (3)	O8—C9—H9B	109.5
O6—N2—C6	118.5 (3)	H9A—C9—H9B	109.5
O5—N2—C6	117.8 (3)	O8—C9—H9C	109.5
O2—C1—O1	126.1 (2)	H9A—C9—H9C	109.5
O2—C1—C2	118.0 (2)	H9B—C9—H9C	109.5
O1—C1—C2	115.9 (2)		
			
O8—Co—O1—C1	87.5 (2)	C1—C2—C3—C4	−179.8 (2)
O8^i^—Co—O1—C1	−92.5 (2)	C2—C3—C4—C5	0.0 (4)
O7—Co—O1—C1	175.6 (2)	C2—C3—C4—N1	179.5 (2)
O7^i^—Co—O1—C1	−4.4 (2)	O3—N1—C4—C5	173.0 (3)
O8—Co—O7—C8	125.9 (2)	O4—N1—C4—C5	−9.2 (5)
O8^i^—Co—O7—C8	−54.1 (2)	O3—N1—C4—C3	−6.5 (5)
O1—Co—O7—C8	34.5 (2)	O4—N1—C4—C3	171.2 (3)
O1^i^—Co—O7—C8	−145.5 (2)	C3—C4—C5—C6	0.2 (4)
O1—Co—O8—C9	−58.0 (3)	N1—C4—C5—C6	−179.3 (3)
O1^i^—Co—O8—C9	122.0 (3)	C4—C5—C6—C7	−0.5 (4)
O7—Co—O8—C9	−147.1 (3)	C4—C5—C6—N2	178.2 (3)
O7^i^—Co—O8—C9	32.9 (3)	O6—N2—C6—C5	177.2 (3)
Co—O1—C1—O2	−6.2 (4)	O5—N2—C6—C5	−3.4 (5)
Co—O1—C1—C2	175.42 (15)	O6—N2—C6—C7	−4.0 (5)
O2—C1—C2—C7	−178.2 (2)	O5—N2—C6—C7	175.4 (3)
O1—C1—C2—C7	0.3 (4)	C5—C6—C7—C2	0.6 (4)
O2—C1—C2—C3	1.6 (4)	N2—C6—C7—C2	−178.1 (3)
O1—C1—C2—C3	−179.9 (2)	C3—C2—C7—C6	−0.3 (4)
C7—C2—C3—C4	0.0 (4)	C1—C2—C7—C6	179.5 (2)

Symmetry codes: (i) −*x*+1, −*y*+1, −*z*+1.

### Hydrogen-bond geometry (Å, °)

**Table:**

*D*—H···*A*	*D*—H	H···*A*	*D*···*A*	*D*—H···*A*
O7—H7o···O2^i^	0.85 (3)	1.840 (15)	2.645 (2)	158 (3)
O8—H8o···O2^ii^	0.85 (3)	1.817 (10)	2.662 (2)	179 (3)

Symmetry codes: (i) −*x*+1, −*y*+1, −*z*+1; (ii) *x*−1, *y*, *z*.
